# Burden of Malaria and Dengue Across Global, Asian, and Chinese Populations Based on GBD 2021 Data: A Quantitative Assessment of Importation Risks to China

**DOI:** 10.3390/v18060690

**Published:** 2026-06-22

**Authors:** Ning Jiang, Weichao Liu, Huifang Zhou, Xianlin Zhan, Xue’e Dai, Wei Yan, Jianhua Yin

**Affiliations:** 1 Department of Laboratory Medicine, Naval Medical Centre, Naval Medical University, Shanghai 200052, China; jiangning990921@163.com (N.J.); 13671552109@163.com (H.Z.); zxlgqc799@163.com (X.Z.); 18317078768@163.com (X.D.); 2 Department of Epidemiology, China Medical University, Shenyang 110122, China; lwc24474169@163.com; 3 Department of Epidemiology, Naval Medical University, Shanghai 200433, China; 4 Key Laboratory of Biological Defense, Ministry of Education, Shanghai 200433, China; 5 Shanghai Key Laboratory of Medical Bioprotection, Shanghai 200433, China

**Keywords:** malaria, dengue, Global Burden of Disease Study (GBD) 2021, quantification of input risk

## Abstract

Background: Malaria and dengue continue to pose significant public health challenges in Asia, with differing temporal trends and regional distributions. However, comparative and long-term assessments of their disease burden and future trajectories remain limited. Methods: Using Global Burden of Disease Study 2021 data, we estimated age-standardized incidence rates (ASIR), disability-adjusted life years (DALYs-ASR), and estimated annual percentage changes (EAPCs) for global, Asian, and Chinese populations by age, sex, and socio-demographic index (SDI). Correlations with SDI and population density were analyzed, and an importation risk index for China was developed. Future trends to 2030 were projected using Bayesian age-period-cohort modeling. Findings: From 1990 to 2021, dengue ASIR increased globally and in China, particularly in middle-SDI regions, whereas malaria ASIR and DALYs-ASR declined substantially, with the most pronounced reductions observed in China. Dengue DALYs-ASR were highest among children under five, while incidence peaked in adolescents; malaria burden was concentrated in young children and young adults. Sex-specific differences were observed, with higher dengue incidence in females but greater DALY rates in males. Geographically, Southeast Asian countries contributed most to the estimated importation risk for both diseases. Projections indicate continued increases in dengue burden through 2030, alongside further declines in malaria. Conclusions: Malaria and dengue exhibit divergent epidemiological patterns across Asia, with declining malaria burden but rising dengue incidence. These findings highlight the need for differentiated control strategies, strengthened regional collaboration, and enhanced surveillance of cross-border transmission.

## 1. Introduction

Neglected tropical diseases (NTDs) and malaria remain major public health challenges across Asia, influenced by diverse climates, ecological gradients, and uneven socioeconomic development [1]. Among NTDs, malaria and dengue are the most widespread and consequential vector-borne infections, placing disproportionate strain on health systems and vulnerable populations. Despite notable advances in prevention and control, both diseases persist due to complex transmission dynamics, substantial cross-border mobility, and gaps in health infrastructure [2,3].

Over the past three decades, the global burden of dengue has increased markedly. Estimates from the Global Burden of Disease Study 2021 (GBD 2021) indicate that incident cases rose from approximately 26.45 million in 1990 to 58.96 million in 2021, accompanied by parallel increases in deaths and disability-adjusted life years (DALYs). Previous studies have consistently identified Tropical Latin America, South Asia and Southeast Asia as high-burden regions, with particularly rapid growth observed in low- and middle-Socio-demographic Index (SDI) settings [4,5].

In contrast, malaria has experienced substantial long-term declines in incidence and mortality following intensified global control efforts since the early 2000s. However, this progress has slowed in recent years, with evidence of stagnation and resurgence during and after the COVID-19 pandemic. According to the World Health Organization (WHO), global malaria cases reached 249 million in 2022 and 263 million in 2023, with mortality remaining above pre-pandemic levels [6]. In Asia, residual transmission persists in specific ecological and geographic settings, particularly in border and rural areas, posing continued risks of reintroduction and cross-border spread [7]. Despite these contrasting trends, comprehensive and comparative assessments of dengue and malaria burdens across Asia, particularly incorporating long-term trajectories, population heterogeneity, and future projections, remain limited.

To address this gap, the present study systematically compared the long-term disease burden, demographic patterns, future trajectories, and potential cross-border transmission risks of dengue and malaria across Asia and China. This study aimed to provide evidence for differentiated prevention and control strategies and to support the sustainability of malaria elimination alongside intensified dengue prevention efforts.

## 2. Materials and Methods

### 2.1. Data Sources

This study utilized data from the GBD 2021 to estimate age-standardized incidence rate (ASIR), age-standardized DALYs (DALYs-ASR), and their corresponding 95% uncertainty intervals (UIs) for malaria and dengue across global and Asian countries. Additional demographic data, including sex, age, and population size, were also extracted to estimate disease burden risks (https://vizhub.healthdata.org/gbd-results/) (accessed on 10 May 2025). Detailed methodological descriptions for GBD 2021 have been published previously [8].

The SDI was included as a key covariate. SDI is a composite measure based on per capita income, mean years of schooling in the population aged 15 years or older, and total fertility rate in women younger than 25 years. Each country was categorized into one of five SDI quintiles (low, low–middle, middle, high–middle, and high). Country-specific thresholds observed in 2021 were used to derive continuous SDI values ranging from 0 to 1, reflecting the overall socio-demographic development status of each location. To better illustrate the distribution of socio-demographic development across the study countries, we extracted the 2021 SDI values for 49 Asian countries included in the analysis and visualized their country-specific SDI values and SDI classifications in Supplementary Figure S1.

In GBD 2021, the definitions of malaria and dengue were primarily based on international case definitions and surveillance standards established by the WHO and regional public health agencies such as the Pan American Health Organization (PAHO). These standardized definitions ensured comparability of case detection and reporting across countries. The GBD framework integrated data from WHO, national surveillance systems, published scientific literature, and population-based surveys. Estimates were then generated using Bayesian meta-regression models (e.g., DisMod-MR), which harmonized heterogeneous data sources to produce globally comparable disease burden estimates.

To quantify the importation risk of malaria and dengue into China from other Asian countries, we incorporated multiple indicators, including disease endemicity, international travel volume, bilateral trade, geographical proximity, and health cooperation (Supplementary Table S1). Malaria endemicity status was determined using the World Malaria Report 2024 [9], which defines a country or area as endemic if it has reported at least one indigenous case since 2021. Based on the dengue endemicity information provided by the WHO, Asian countries listed as dengue-endemic were classified as endemic, while those not listed were classified as non-endemic. The overall global prevalence and distribution of dengue fever were further described using WHO-reported data. (https://www.who.int/zh/emergencies/disease-outbreak-news/item/2023-DON498) (accessed on 11 May 2025). To analyze the dengue epidemic in 2024, we selected the “total cases per 100,000 population” for Asian countries and incorporated this indicator into our assessment (https://worldhealthorg.shinyapps.io/dengue_global/) (accessed on 16 May 2025). ASIRs of malaria and dengue were extracted from GBD 2021 as averages for 2019–2021. International inbound arrivals to China were obtained from the United Nations World Tourism Organization (UNWTO) database (https://www.e-unwto.org/doi/book/10.18111/9789284424139) (accessed on 19 May 2025), and bilateral trade volumes were retrieved from the General Administration of Customs of China (averaged for 2019–2021; http://stats.customs.gov.cn/) (accessed on 21 May 2025). Information on health cooperation projects was collected from the Belt and Road Initiative official website (https://www.yidaiyilu.gov.cn/) (accessed on 15 June 2025), and data on direct flight connections were extracted from international travel portals (https://www.ctrip.com/) (accessed on 17 June 2025). Geographic adjacency and capital-to-capital distances were determined using global maps. These variables were synthesized into a composite risk index. The composite risk index was normalized to a 0–100 linear scale to facilitate cross-country comparison and policy interpretation. This index was designed as a standardized comparative metric rather than a direct probabilistic estimate of importation risk. Given that the included indicators differed substantially in scale, units, and distributions, linear normalization provided a consistent and transparent framework for integrating heterogeneous epidemiological, mobility-related, economic, and geographic factors. A higher score therefore indicates a higher relative priority for importation risk monitoring and prevention, rather than an absolute probability of imported transmission.

In GBD, incidence and DALYs are reported as crude rates per 100,000 population, while ASRs are calculated using the GBD standard population age structure. To evaluate temporal trends, we calculated the estimated annual percentage change (EAPC) for both ASIR and DALYs-ASR for malaria and dengue. We further assessed correlations between EAPC and SDI as well as population density. Age- and sex-specific population data from GBD were also analyzed to assess subgroup differences and to project the burden of malaria and dengue from 2022 to 2030. In addition, reported case numbers of malaria and dengue from the China Health Statistical Yearbook (1990–2021) were converted into crude incidence rates and compared with GBD estimates.

### 2.2. Statistical Analysis

To ensure comparability across variables in the composite risk index, min–max normalization was applied to rescale data to a 0–1 range. Specifically, incidence rate, international arrivals, and average bilateral trade volume were normalized using positive standardization:

$$Z=\frac{X-X_{min}}{X_{max}-X_{min}}$$

Capital-to-capital distance was normalized using negative standardization:

$$Z=\frac{X_{max}-X}{X_{max}-X_{min}}$$

Weights for each indicator were determined through expert consultation to reflect the relative importance of each factor in capturing the potential risk of imported malaria and dengue transmission to China. Indicators directly related to disease burden and cross-border mobility or economic exchange were assigned higher weights, while contextual factors received lower weights. Accordingly, the 3-year average ASIR and endemicity status were each assigned 15%, annual inbound arrivals to China 30%, and the 3-year average bilateral trade volume 20%, whereas geographic adjacency and capital-to-capital distance were assigned 5% each, and public health cooperation projects 10%. This weighting scheme integrates epidemiological, demographic, economic, and geographical dimensions, emphasizing the most influential drivers of importation risk while still accounting for relevant contextual factors, and provides a transparent, expert-informed framework for constructing the composite risk index.

For correlation analyses, Pearson correlation coefficients (R) and corresponding *p*-values were calculated to evaluate the associations between the EAPC of ASIR and DALYs-ASR with SDI and population density. Population density was log-transformed using log10(population density + 1) to reduce the influence of extreme outliers. For visualization in Supplementary Figure S1, the transformed population density was further stratified into six quantile-based groups, from Q1 to Q6.

To project the ASIR of malaria and dengue through 2030, we employed a Bayesian age-period-cohort (BAPC) model implemented in the R package BAPC (version 4.4.3). This model combined GBD 1990–2021 data with population estimates and standard projection variants from the World Population Prospects 2021. Parameters were estimated using the Integrated Nested Laplace Approximation (INLA) method.

### 2.3. Software

All statistical analyses and visualizations were conducted using R software (version 4.4.3). All tests were two-sided, and a *p*-value < 0.05 was considered statistically significant.

## 3. Results

### 3.1. Temporal Trends Across Regions

**Figure 1:**
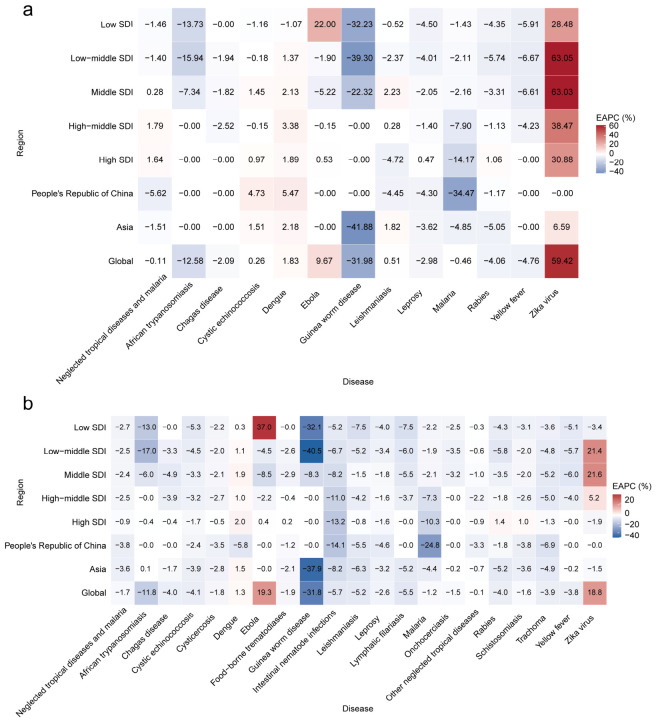
Estimated annual percentage change (EAPC) in age-standardized incidence rate (ASIR) (**a**) and age-standardized disability-adjusted life years rate (DALYs-ASR) (**b**) for various tropical diseases across the global Asia, China, and Socio-demographic Index (SDI) regions, 1990–2021.

From 1990 to 2021, we analyzed tropical diseases across five SDI regions, as well as globally, in Asia, and in China (excluding diseases with missing data). The EAPC of ASIR and DALYs ASR are shown in Figure 1a,b. Overall, the EAPC of neglected tropical diseases remained stable at relatively low levels across all regions.

At the disease-specific level, Guinea worm disease and malaria showed markedly declining ASIR EAPC, with reductions of up to 40% in some regions; similar downward trends were observed for DALYs-ASR EAPC. In contrast, dengue exhibited modest increases in ASIR EAPC across all SDI regions except low SDI, with China showing a steeper rise than the global average. However, dengue DALYs-ASR EAPC continued to decline. Notably, malaria-related declines were more pronounced in China than in other regions. These contrasting trends highlight malaria and dengue as representative vector-borne diseases for further analysis: malaria with declining burden but ongoing importation risk, and dengue with rising incidence and growing public health relevance, particularly in China. This justifies focusing on these two diseases in subsequent analyses.

### 3.2. Global Burden of Dengue and Malaria

In 2021, the global DALYs-ASR and ASIR for dengue were 27.76 (95% UI: 14.21–41.65) and 752.04 (95% UI: 196.33–1363.35) per 100,000 persons, respectively. Compared to 1990 values (DALYs-ASR: 21.63; 95% UI 15.09–26.92; ASIR: 481.85; 95% UI: 70.76–946.29), DALYs-ASR and ASIR both increased, with corresponding EAPCs of 1.33 (95% UI: 1.10–1.57) and 1.83 (95% UI: 1.58–2.08), respectively (Supplementary Table S2). For malaria, the 2021 DALYs-ASR and ASIR were 806.00 (95% UI: 318.93–1570.18) and 3485.27 (95% UI: 2804.46–4435.69) per 100,000, respectively. Both Values were lower than those in 1990 (DALYs-ASR: 965.67; 95% UI: 502.56–1898.07; ASIR: 3689.81;95% UI: 3133.05–4440.58), consistent with negative EAPCs of −1.17 (95% UI: −1.57 to −0.77) for DALYs-ASR and −0.46 (95% UI: −0.65 to −0.27) for ASIR (Supplementary Table S3).

### 3.3. Burden Patterns Across SDI Regions

By SDI regions, the highest burden of dengue in 1990 was observed in the low-middle SDI region, with an ASIR of 802.75 per 100,000 (95% UI: 79.39–1725.04) and DALYs-ASR of 35.92 per 100,000 (95% UI: 24.95–47.94). By 2021, the most pronounced increase occurred in the middle SDI group, where the ASIR reached 1269.27 per 100,000 (95% UI: 437.36–2268.00) and the DALYs-ASR was 48.78 per 100,000 (95% UI: 27.32–71.02) (Supplementary Table S2). For malaria, the greatest burden in 1990 was in low SDI countries, with an ASIR of 17,529.12 per 100,000 (95% UI: 14,625.05–20,999.26) and a DALYs-ASR of 4811.47 per 100,000 (95% UI: 2580.65–8569.12). Although these values declined by 2021, the low SDI group continued to exhibit the highest rates (ASIR: 11,883.52; 95% UI: 9755.64–14,691.05; DALYs-ASR: 2869.17; 95% UI: 1107.42–5683.87), supported by negative EAPCs of −1.43 (95% UI: −1.60 to −1.27) for ASIR and −2.17 (95% UI: −2.45 to−1.89) for DALYs-ASR (Supplementary Table S3).

### 3.4. National-Level Heterogeneity

At the national level, Indonesia reported the highest dengue DALYs-ASR in 2021 (279.79 per 100,000; 95% UI: 170.93–404.43), which remained comparable to its 1990 level (282.38 per 100,000; 95% UI: 170.88–457.73), reflecting a modest EAPC of 0.28 (95% UI: 0.12–0.43). In contrast, Singapore exhibited the highest ASIR for dengue, rising from 7258.06 (95% UI: 1064.77–18,986.36) in 1990 to 8714.74 (95% UI: 1927.54–20,177.45) in 2021, with an EAPC of 1.33 (95% UI: 0.23–2.44). The Philippines showed the highest EAPC of 6.93 (95% UI: 6.43–7.43) for dengue ASIR (Supplementary Table S2).

For malaria, Armenia experienced the largest decline in DALYs-ASR (EAPC: −29.99; 95% UI: −39.85 to −18.50), followed by Bhutan (EAPC: −26.20; 95% UI: −28.17 to −24.17). Several countries achieved zero malaria ASIR between 1990 and 2021, with the most substantial decline observed in the Syrian Arab Republic (EAPC: −40.02; 95% UI: −51.72 to −25.47), trailed by Iraq, Sri Lanka, and Bhutan (all with EAPCs below −30%) (Supplementary Table S3). Notably, China demonstrated effective disease control, evidenced by a dengue ASIR EAPC of 5.47 (95% UI: 4.54–6.40) and a malaria ASIR EAPC of −26.89 (95% UI: −32.73 to −20.55) (Supplementary Tables S2 and S3).

### 3.5. Age- and Sex-Specific Patterns

**Figure 2:**
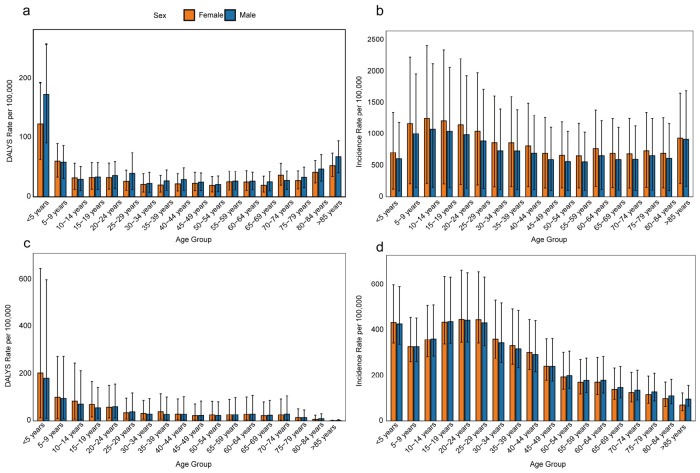
Age- and sex-specific incidence and disability-adjusted life years (DALYs) for malaria and dengue in 2021: (**a**) dengue DALYs, (**b**) dengue incidence, (**c**) malaria DALYs, (**d**) malaria incidence.

Age- and sex-specific analyses revealed distinct burden patterns for dengue and malaria across 18 age groups (ranging from 0 to ≥85 years). For dengue, the highest DALYs rate was observed in the 0–4 years group (male: 174.18; 95% UI: 91.65–258.44; female: 123.81; 95% UI: 63.47–193.56). Incidence rate peaked in the 10–14 years group (male: 1079.48; 95% UI: 155.77–2121.04; female: 1250.49; 95% UI: 215.99–2409.19), with a secondary increase among those aged ≥85 years (Incidence rate: male 914.63; 95% UI: 169.99–1694.47; female 932.51; 95% UI: 214.11–1657.49; DALYs rate: male 68.10; 95% UI: 41.06–95.01; female 53.30; 95% UI: 34.85–73.96) (Figure 2a,b). For malaria, the highest DALYs rate was similarly found in the 0–4 years group (male: 181.81; 95% UI: 11.52–600.87; female: 205.33; 95% UI: 13.35–647.47). However, the Incidence rate displayed a different pattern, with peaks in the 0–4, 15–19, 20–24, and 25–29 years groups. The highest Incidence rate occurred in the 20–24 years group (male: 443.99; 95% UI: 346.97–652.24; female: 447.39; 95% UI: 346.22–663.17) (Figure 2c,d).

### 3.6. Associations Between EAPC, SDI, and Population Density

Pearson correlation analyses revealed no significant associations between the EAPC in ASIR of dengue and the SDI or population density (Supplementary Figure S2a,b). However, the EAPC in DALYs-ASR for dengue exhibited weak negative correlations with both SDI (R = −0.312) and population density (R = −0.225) (Supplementary Figure S2c,d). Regarding malaria, a weak negative correlation was observed between ASIR EAPC and SDI (R = −0.252, Supplementary Figure S2e), but no significant association was found with population density (Supplementary Figure S2f). Similarly, the EAPC in malaria DALYs-ASR showed a weak negative correlation with SDI, but no correlation with population density (Supplementary Figure S2g,h).

### 3.7. Importation Risk Assessment

We further evaluated the importation risk of malaria across 36 Asian countries with sufficient data by calculating standardized and weighted composite scores. Countries were categorized into quartiles based on importation risk levels. Lebanon demonstrated the lowest risk (0), followed by Jordan (0.09), whereas Viet Nam (100) and the Republic of Korea (92.80) were associated with the highest risks. The remaining countries exhibited intermediate risks at varying levels (Table 1). Similarly, we also evaluated 21 countries with a high risk of dengue importation to China, using the same method as for malaria. Among these countries, Armenia (0) and Azerbaijan (0.69) had the lowest risk of importation; Vietnam (100) and Myanmar (79.96) had the highest risk; and other countries had varying degrees of risk (Table 2).

### 3.8. Projected Trends of Dengue and Malaria Incidence Through 2030

**Figure 3:**
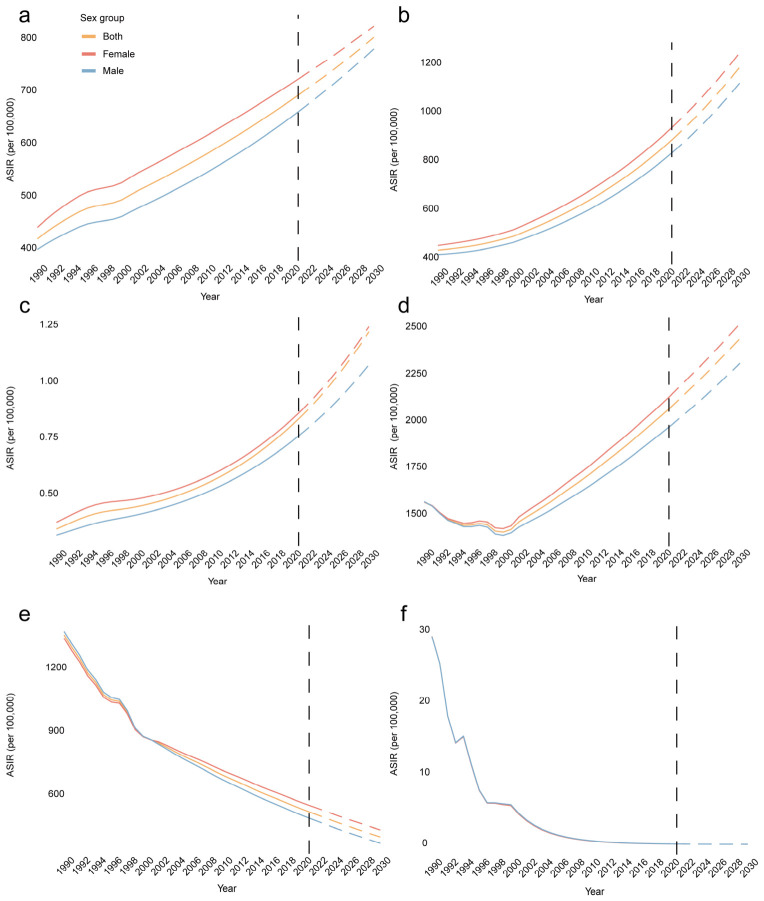
Temporal trends in age-standardized incidence rate (ASIR) of malaria and dengue from 1990 to 2030 at the global level, in Asia, and in China: (**a**) Global dengue, (**b**) Asian dengue, (**c**) Chinese dengue, (**d**) Global malaria, (**e**) Asian malaria, (**f**) Chinese malaria.

Using a Bayesian age-period-cohort model, we projected the ASIR of dengue and malaria from 2022 to 2030 at the global, Asian, and Chinese levels. For dengue, ASIR is projected to increase steadily, peaking in 2030. Females are expected to have the highest rates across all regions: 824.54 per 100,000 globally, 1246.62 per 100,000 in Asia, and 1.25 per 100,000 in China (Figure 3a–c). For malaria, trends varied by region. Globally, ASIR is predicted to increase, reaching a peak of 2521.52 per 100,000 among females by 2030 (Figure 3d). In Asia, however, a decline is projected, with the lowest rate observed in males (371.29 per 100,000) by 2030 (Figure 3e). In China, ASIR is expected to remain extremely low and stable for both sexes, with an overall rate of 0.0071 per 100,000 by 2030 (Figure 3f).

## 4. Discussion

Our findings underscore that malaria and dengue remain critical public health threats in tropical disease control. Between 1990 and 2021, dengue exhibited considerable spatial variation in ASIR and EAPC across most countries and regions, with a clear overall increasing trend. Although malaria incidence has declined worldwide, elevated ASIR levels persist in several countries. Subgroup analyses and predictive modelling further identify infants and women as disproportionately affected, highlighting them as priority populations for targeted interventions.

### 4.1. Malaria: Declining Burden, Importation Risk, and Future Trends

In China, malaria elimination was certified by WHO in 2021 following three consecutive years with no indigenous cases. However, recent surveillance data suggest a potential resurgence, with 3157 malaria cases reported in 2024 [10]. This indicates that there is still a relatively high risk of imported malaria cases from neighboring countries into China.

Cross-country comparisons highlight substantial heterogeneity in tropical disease burdens, which warrants closer attention. In the heatmap, malaria shows consistent declines in both ASIR and DALYs-ASR, with the most marked reduction in China- an achievement attributable to effective vector control strategies, including larval source management [11] and adaptive, targeted vector control approaches [12,13,14]. In addition, regional deforestation may also partly contribute to heterogeneous malaria trends, although its effect appears to be time-dependent. Evidence from Lao PDR showed that deforestation around villages was associated with higher malaria incidence in the short term but lower incidence over the longer term, suggesting that land-use change may be one possible ecological factor related to declining malaria transmission in some settings [15]. Sex- and Age-stratified analyses reveal distinct epidemiological patterns. Malaria incidence was highest among young adults, likely due to occupational exposure and outdoor activities. The elevated burden among women of reproductive age also raises concerns regarding mother-to-child transmission risks, consistent with global malaria trends in children [6].

We further quantified malaria importation risks from 36 Asian countries to China, identifying Vietnam, Myanmar, and South Korea as the highest-risk sources, each influenced by distinct factors: endemic transmission and cross-border mobility (Vietnam and Myanmar) and high travel volume (South Korea). These findings support the need for tiered, precision screening strategies, with enhanced diagnostic and surveillance capacity at high-risk points of entry such as land borders with Vietnam and Myanmar, and air hubs with Korea. Additional attention should be targeted at interventions for high-risk groups such as cross-border laborers aged 15–29. Dynamic resource allocation based on updated risk assessments, integrated electronic declarations for lower-risk routes, and coordinated detection–treatment–traceback systems are strongly recommended. Moreover, outbound travelers to high-risk regions should receive tailored preventive guidance. This risk stratification framework provides an evidence-based approach for transitioning from uniform to precision border health management.

According to BAPC-based projections, malaria presents divergent trends: global incidence is projected to increase—driven by setbacks in malaria control during the COVID-19 pandemic [9], constrained access to artemisinin-based therapies [16], climate-facilitated vector proliferation [17], and persistent poverty undermining health systems [18]—while declines are projected in Asia and China. These declines reflect successful elimination certification in China [19] and strengthened preventive and control measures across Asia, including adoption of best practices from low-burden settings [20,21].

### 4.2. Dengue: Increasing Burden, Population Patterns, and Importation Risk

Dengue has recurred in large-scale outbreaks in China, notably in 2014 and 2019, and has demonstrated a steady increase in incidence since 1990. Projections indicate that this upward trend is likely to continue through at least 2030. Heatmap analyses of EAPC indicate that dengue incidence is rising across all SDI categories except low-SDI countries, while DALYs increased across most regions except China. These disparities may be attributed to rapid population growth, climatic conditions conducive to vector proliferation [22], and disproportionate vector exposure in lower-income urban settings [23]. Notably, even high SDI countries report sustained increases in ASIR and DALYs-ASR. This may reflect intensified globalization and international trade, including the spread of vectors via imported used tires [24], as well as dengue importation and local transmission facilitated by international travel [25].

In the stratified analyses by sex and age for dengue, the incidence peaked among adolescents, consistent with prior studies [26]. This predominance in adolescents may be partly related to school-based exposure and contact patterns, as school-aged children and teenagers have been reported to represent a high-risk group for dengue infection [27]. Schools may therefore serve not only as potential settings for dengue-related exposure, but also as important sites for health education and community-based vector control interventions. A previous study reported that the highest DALYs were observed in children under five, particularly infants, with the burden exceeding 333.46 per 100,000 in neonates [28]. This may reflect waning maternal antibodies, which initially provide partial protection but subsequently predispose infants to more severe dengue manifestations, including dengue hemorrhagic fever [29,30]. Furthermore, women exhibited higher dengue incidence rates, whereas men showed higher DALYs burdens, potentially reflecting gender differences in health-seeking behavior and delayed healthcare access among men [31].

Among the 21 countries with a risk of dengue importation, quantification was also conducted. Vietnam and Myanmar were identified as the most severely affected countries. This coincides with the situation of malaria high-risk countries, suggesting that in the future, joint risk control for both diseases can be implemented to help formulate new prevention and control strategies.

The BAPC prediction shows that dengue incidence and DALYs-ASR will continue to increase globally, across Asia, and in China until 2030. Improved diagnostic sensitivity, climate change, and expanded geographic suitability for vector habitats are key contributors [32,33].

Several limitations should be noted. First, data were primarily sourced from GBD 2021, which, like all GBD analyses, is subject to uncertainty due to data availability, diagnostic variability, and reporting quality across countries [34]. Underreporting and inconsistent case definitions may affect dengue estimates. Although we identified sex-based differences in dengue metrics, the underlying mechanisms remain unclear and warrant further study [4]. Comparisons with China Health Statistical Yearbook data also revealed discrepancies, likely influenced by COVID-19 and concurrent infectious diseases, which disrupted reporting systems. Notably, national yearbook data captured the sharp incidence spikes during the 2014 and 2019 dengue epidemics, which were not reflected in GBD estimates (Supplementary Figure S3a), while malaria incidence estimates from GBD appeared closer to real-world trends over time (Supplementary Figure S3b).

In addition, the importation risk index developed in this study may be influenced by assumptions related to population mobility. Specifically, populations in highly endemic but economically disadvantaged regions may have limited capacity for international travel, which could lead to a potential overestimation of importation risk from these settings. Furthermore, the current analysis did not incorporate observed importation case data for validation. Future studies integrating real-world surveillance data will be important for evaluating the predictive performance of the index and further refining the model.

## 5. Conclusions

Over the past three decades, the burden of dengue and malaria has continued to increase globally. These findings highlight the urgency of transnational collaboration, experience sharing, and targeted prevention strategies to reduce burdens in high-risk regions and populations. Furthermore, our proposed quantitative approach for assessing importation risk offers a practical tool to support entry screening tailored to countries or regions, while accounting for sex- and age-specific differences, thereby improving the efficiency of time and resource allocation. Collectively, this study provides evidence to strengthen health systems, facilitate intersectoral collaboration, and optimize resource integration, supporting efforts to reduce the burden of dengue and malaria in China, Asia, and worldwide and contributing to sustainable development goals.

## Figures and Tables

**Table 1:** Risk Index of Malaria Importation to China by Asian Countries.

Scope	Country	Value
0–25	Lebanon	0.00
	Jordan	0.09
	Kuwait	1.78
	Timor-Leste	1.81
	Bahrain	18.09
	Armenia	18.40
	Maldives	18.45
	Qatar	18.71
	Azerbaijan	18.98
	Sri Lanka	19.81
	Turkmenistan	20.08
	Uzbekistan	21.46
	Saudi Arabia	21.55
	Bhutan	22.37
25–50	Democratic People’s Republic of Korea	27.40
	Iran	28.65
	Singapore	29.01
	Tajikistan	29.49
	Brunei Darussalam	29.64
	Kyrgyzstan	30.51
	Malaysia	41.99
	Afghanistan	42.41
	India	48.25
	Bangladesh	49.41
	Cambodia	49.43
50–75	Yemen	50.85
	Thailand	56.49
	Nepal	56.95
	Lao People’s Democratic Republic	58.54
	Japan	59.98
	Indonesia	62.70
	Pakistan	63.86
	Philippines	64.91
75–100	Myanmar	92.41
	Republic of Korea	92.80
	Viet Nam	100.00

**Table 2:** Risk Index of Dengue Importation to China by Asian Countries.

Scope	Country	Value
0–25	Armenia	0.00
	Azerbaijan	0.69
	Turkmenistan	1.98
	Uzbekistan	3.61
	Tajikistan	13.06
	Kyrgyzstan	14.27
	Afghanistan	16.80
25–50	Saudi Arabia	27.90
	India	34.72
	Cambodia	35.64
	Bangladesh	37.71
	Sri Lanka	38.93
	Pakistan	43.11
	Thailand	47.18
	Lao People’s Democratic Republic	47.70
	Maldives	47.79
	Nepal	48.71
50–75	Malaysia	67.03
	Singapore	72.93
75–100	Myanmar	79.96
	Viet Nam	100.00

## Data Availability

The datasets generated and/or analyzed during the current study are available in the GBD 2021. Publicly available datasets were analyzed in the current study. The data can be found here: http://ghdx.healthdata.org/gbd-results-tool (accessed on 10 May 2025). The analyzed data will be shared upon reasonable request to the corresponding author.

## Supplementary Materials

The following supporting information can be downloaded at: https://www.mdpi.com/article/10.3390/v18060690/s1, Table S1. Risk index evaluation parameters and weights. Table S2. ASIR and DALYs-ASR of Dengue in Global, Asian, and SDI regions in 1990 and 2021. Table S3. ASIR and DALYs-ASR of Malaria in Global, Asian, and SDI regions in 1990 and 2021. Figure S1. Country-level SDI values and classifications of the 49 Asian countries included in this study. Figure S2. Correlation analyses between estimated annual percentage change (EAPC) and Socio-demographic Index (SDI) or population density. Figure S3. Comparison of incidence rate per 100,000 people in China Health Yearbook and GBD from 1990 to 2021.

## Abbreviations

The following abbreviations are used in this manuscript:

ASIR	Age-standardized incidence rate
ASR	Age-standardized rate
BAPC	Bayesian age-period-cohort
COVID-19	Corona Virus Disease 2019
DALYs	Disability-adjusted life-years
EAPC	Estimated annual percentage change
GBD	Global Burden of Disease Study
INLA	Integrated Nested Laplace Approximation
NTDs	Neglected tropical diseases
PAHO	Pan American Health Organization
SDI	Socio-demographic index
UI	Uncertainty interval
UNWTO	United Nations World Tourism Organization
WHO	World Health Organization
