# Supplementary material for: Burden of Malaria and Dengue Across Global, Asian, and Chinese Populations Based on GBD 2021 Data: A Quantitative Assessment of Importation Risks to China
Source: Viruses. 2026 Jun 22;18(6):690. doi: 10.3390/v18060690 (PMC13307693; doi:10.3390/v18060690)
Supplement: Supplementary file 1 [file viruses-18-00690-s001.zip › viruses-4354188-supplementary.pdf]

Table S1. Risk index evaluation parameters and weights

Indicator	Description	Data source	Processing methods
ASIR	Average ASIR value in the past 3 years (per 100 000 people)	GBD	Min-Max 0-1 Standardization Method
Malaria epidemic level	Prevalence=1 Non prevalence=0	World malaria report 2024	Directly assign points
Number of arrivals	Annual number of arrivals in 2019	UNWTO	Min-Max 0-1 Standardization Method
Annual bilateral trade volume	Annual average bilateral trade volume from 2019 to 2021	GACC	Min-Max 0-1 Standardization Method
Direct flights	Direct flight=1 No direct flight=0	Ctrip Travel Network	Directly assign points
Bordering with China	Yes=1 No=0	World Map	Directly assign points
Distance between countries	The straight-line distance between the capital and Beijing	World Map	Max-Min 0-1 Standardization Method
Bilateral cooperation	Yes=1 No=0	Belt and Road Portal	Directly assign points

ASIR Age-Standardized Incidence Rate, *UNWTO* United Nations World Tourism Organization, *GACC* General Administration of Customs of the People's Republic of China

Table S2. ASIR and DALYs-ASR of Dengue in Global, Asian, and SDI regions in 1990 and 2021

	DALYs-ASR			ASIR		
	1990	2021	EAPC	1990	2021	EAPC
	Age-standard rate	Age-standard rate	(95%UI)	Age-standard rate	Age-standard rate	(95%UI)
Afghanistan	0.04(0.02, 0.13)	0.05(0.01, 0.17)	0.70(0.54, 0.86)	1.41(0.07, 8.65)	2.00(0.04, 13.15)	1.65(1.32, 1.99)
Bahrain	0.24(0.11, 0.44)	0.12(0.05, 0.23)	-2.78(-3.04, -2.52)	0.00(0.00, 0.00)	0.00(0.00, 0.00)	0.00(0.00, 0.00)
Bangladesh	2.91(0.39, 7.80)	4.82(1.13, 10.87)	2.03(1.57, 2.51)	242.08(5.31, 600.71)	425.31(70.01, 827.39)	1.97(1.79, 2.14)
Bhutan	5.93(2.21, 10.89)	11.27(3.49, 24.39)	2.71(1.83, 3.59)	33.26(0.28, 103.11)	52.04(1.38, 146.69)	2.03(1.57, 2.51)
Brunei Darussalam	5.46(1.71, 13.28)	4.61(1.51, 10.62)	0.35(-0.33, 1.03)	46.13(3.11, 153.46)	130.21(34.37, 322.56)	4.68(3.56, 5.81)
Cambodia	18.79(8.55, 38.77)	20.47(8.27, 40.50)	-0.38(-2.36, 1.64)	186.02(36.52, 446.16)	249.2(51.84, 544.45)	1.19(0.83, 1.55)
China	0.32(0.20, 0.45)	0.05(0.02, 0.10)	-5.81(-6.30, -5.32)	0.38(0.01, 1.36)	2.01(0.55, 6.23)	5.47(4.54, 6.40)
India	42.24(21.16, 67.68)	62.77(21.96, 110.46)	1.87(1.62, 2.12)	1317.99(13.42, 2809.06)	1997.18(100.91, 4261.45)	1.55(1.49, 1.61)
Indonesia	282.38(170.88, 457.73)	279.79(170.93, 404.43)	0.28(0.12, 0.43)	912.38(108.00, 3055.06)	906.32(356.12, 1850.51)	0.21(0.06, 0.36)
Japan	0.04(0.03, 0.04)	0.01(0.00, 0.01)	-3.69(-4.26, -3.10)	0.00(0.00, 0.00)	0.00(0.00, 0.00)	0.00(0.00, 0.00)

Jordan	0.14(0.05, 0.40)	0.18(0.04, 0.63)	3.54(1.04, 6.09)	9.06(0.29, 34.95)	14.74(0.48, 52.63)	5.07(2.03, 8.20)
Kuwait	1.08(0.23, 3.21)	0.59(0.03, 2.26)	0.36(-2.03, 2.81)	88.51(3.61, 297.04)	57.30(3.83, 205.42)	1.09(-1.41, 3.65)
Lao People's Democratic Republic	12.67(5.21, 26.28)	14.39(5.07, 28.34)	1.01(-0.25, 2.29)	163.44(2.30, 520.10)	323.91(81.57, 669.51)	1.94(0.66, 3.24)
Lebanon	0.22(0.02, 0.78)	0.34(0.02, 1.22)	4.16(1.52, 6.87)	20.51(1.47, 69.58)	32.11(1.51, 112.07)	4.16(1.49, 6.90)
Malaysia	51.82(27.27, 87.47)	57.98(31.11, 102.26)	0.31 (-0.51, 1.14)	873.13(50.77, 2476.59)	2586.69(976.79, 6552.92)	5.38(4.49, 6.29)
Maldives	72.37(22.92, 169.08)	86.34(27.26, 229.39)	1.39(0.70, 2.08)	344.26(0.00, 1555.32)	5095.16(0.00, 18143.76)	10.54(9.00, 12.11)
Myanmar	56.72(24.46, 125.30)	49.86(19.22, 91.40)	-0.37(-0.51, -0.22)	77.50(14.67, 190.35)	79.26(17.15, 174.81)	2.03(1.03, 3.04)
Nepal	47.49(28.69, 80.75)	52.12(23.00, 97.99)	0.65(0.44, 0.86)	502.04(0.74, 1442.38)	887.98(8.79, 2609.38)	2.13(1.94, 2.33)
Oman	24.66(13.93, 39.91)	13.84(7.42, 25.31)	-1.64(-1.78, -1.50)	12.96(0.14, 113.62)	22.61(0.11, 126.97)	1.82(1.22, 2.43)
Pakistan	16.78(3.89, 39.33)	29.82(9.91, 60.29)	2.27(2.12, 2.42)	960.44(5.10, 2720.46)	1125.50(22.41, 3092.40)	0.56(0.51, 0.60)
Palestine	0.37(0.17, 0.88)	0.34(0.12, 0.99)	1.48(-0.19, 3.18)	16.32(0.85, 54.71)	19.76(1.19, 73.23)	2.86(0.41, 5.37)
Philippines	62.86(50.97, 76.85)	126.60(95.17, 164.20)	3.68(3.13, 4.23)	164.37(10.08, 419.90)	1111.20(265.17, 3936.42)	6.93(6.43, 7.43)
Qatar	0.13(0.05, 0.24)	0.07(0.02, 0.13)	-2.17(-2.48, -1.85)	0.00(0.00, 0.00)	0.00(0.00, 0.00)	0.00(0.00, 0.00)
Republic of Korea	0.01(0.00, 0.01)	0.00(0.00, 0.00)	-7.65(-8.56, -6.72)	0.00(0.00, 0.00)	0.00(0.00, 0.00)	0.00(0.00, 0.00)
Saudi Arabia	0.32(0.12, 0.68)	0.79(0.30, 1.46)	4.75(3.70, 5.81)	1.23(0.27, 3.22)	4.82(1.37, 14.25)	4.71(3.97, 5.46)
Singapore	70.73(8.02, 193.43)	85.30(15.20, 216.32)	1.39(0.27, 2.51)	7258.06(1064.77, 18986.36)	8714.74(1927.54, 20177.45)	1.33(0.23, 2.44)
Sri Lanka	47.93(31.09, 75.90)	59.54(35.34, 109.82)	2.16(0.92, 3.43)	467.35(26.21, 1368.07)	2106.45(552.08, 7025.59)	6.09(5.41, 6.77)
Syrian Arab Republic	0.05(0.02, 0.14)	0.05(0.02, 0.15)	2.12 (-0.34, 4.65)	3.06(0.23, 11.78)	3.70(0.31, 12.24)	3.01(0.06, 6.05)
Taiwan (Province of China)	1.58(0.16, 5.66)	2.47(0.59, 6.86)	3.03(2.32, 3.74)	154.00(15.24, 538.66)	147.88(9.94, 536.03)	0.00(-0.27, 0.27)
Thailand	45.33(26.91, 72.57)	20.79(12.53, 32.24)	-2.24(-2.68, -1.79)	358.80(75.61, 739.59)	630.40(174.78, 1381.13)	3.95(3.14, 4.76)
Timor-Leste	15.40(7.47, 35.48)	19.96(9.84, 36.76)	0.98(-0.48, 2.47)	79.35(6.19, 284.69)	221.18(58.52, 566.70)	3.70(2.96, 4.45)
Viet Nam	12.18(4.99, 24.96)	16.84(6.06, 34.57)	0.68(0.02, 1.35)	606.03(108.05, 1505.97)	1118.13(415.08, 700.97)	1.56(1.32, 1.80)
Yemen	0.16(0.02, 0.93)	0.26(0.03, 1.39)	4.17(0.80, 7.64)	13.5(0.55, 90.46)	23.76(2.37, 126.03)	1.79(1.63, 1.96)
Asia	33.01(24.04, 40.48)	44.53(23.29, 65.36)	1.47(1.26, 1.68)	484.77(25.08, 1011.28)	862.40(168.28, 1638.55)	2.18(2.08, 2.29)
Global	21.63(15.09, 26.92)	27.76(14.21, 41.65)	1.33(1.10, 1.57)	481.85(70.76, 946.29)	752.04(196.33, 1363.35)	1.83(1.58, 2.08)

High SDI	0.42(0.09, 1.07)	0.58(0.14, 1.39)	1.96(1.04, 2.90)	38.03(5.47, 92.83)	54.64(12.22, 119.21)	1.89(0.93, 2.87)
High-middle SDI	8.92(6.20, 12.61)	11.39(6.67, 16.03)	1.00(0.67, 1.33)	98.43(12.21, 263.08)	215.66(91.61, 372.30)	3.38(2.98, 3.79)
Middle SDI	33.64(21.43, 42.85)	48.78(27.32, 71.02)	1.87(1.58, 2.16)	782.97(51.81, 1725.40)	1269.27(437.36, 2268.00)	2.13(1.83, 2.43)
Low-middle SDI	35.92(24.95, 47.94)	43.35(19.69, 69.74)	1.06(0.86, 1.25)	802.75(79.39, 1725.04)	1117.70(123.23, 2373.55)	1.37(1.18, 1.56)
Low SDI	11.84(8.11, 16.08)	12.34(4.44, 22.08)	0.34(0.05, 0.64)	431.07(242.88, 648.51)	368.03(11.69, 884.71)	-1.06(-1.71, -0.42)

ASIR age-standardized incidence rate, *ASR* age-standardized rate, *DALY* disability-adjusted life year, *EAPC* estimated annual percentage change, *UI* uncertainty interval

Table S3. ASIR and DALYs-ASR of Malaria in Global, Asian, and SDI regions in 1990 and 2021

	DALYs-ASR			ASIR		
	1990	2021	EAPC (95%UI)	1990	2021	EAPC (95%UI)
	Age-standard rate	Age-standard rate		Age-standard rate	Age-standard rate	
Afghanistan	91.84(41.95, 204.02)	28.87(15.34, 42.11)	-3.84(-6.65, -0.95)	8460.85(6449.69 , 10824.53)	772.70(624.86, 950.36)	-5.59(-6.98, -4.19)
Armenia	0.00(0.00, 0.00)	0.00(0.00, 0.00)	-29.99(- 39.85, - 18.50)	0.00(0.00, 0.00)	0.00(0.00, 0.00)	-12.27(- 39.39, 26.97)
Azerbaijan	1.78(1.55, 2.04)	0.00(0.00, 0.00)	-15.55(- 19.88, - 11.00)	2.17(1.42, 3.11)	0.00(0.00, 0.00)	-21.25(- 32.13, - 8.62)
Bangladesh	659.45(64.20, 2860.12)	20.83(5.74, 40.71)	-8.80(- 10.41, - 7.16)	1383.96(377.42, 3735.34)	34.45(30.36, 38.98)	-10.09(- 11.54, - 8.62)
Bhutan	5885.44(951.00, 16 344.28)	2.96(2.13, 4.16)	-26.20(- 28.17, - 24.17)	76391.27(29840. 23, 166794.1)	8.20(7.16, 9.49)	-30.40(- 32.46, - 28.29)
Cambodia	722.90(346.19, 1585.06)	5.74(4.12, 7.90)	-8.70(- 11.29, - 6.04)	7227.5(6152.88, 8402.22)	95.14(79.71, 111.96)	-7.96(-9.49, -6.40)
China	5.18(0.26, 56.53)	0.00(0.00, 0.00)	-19.85(- 21.34, - 18.34)	0.00(0.00, 0.00)	0.00(0.00, 0.00)	-26.89(- 32.73, - 20.55)
Democratic People's Republic of Korea	0.00(0.00, 0.00)	0.88(0.62, 1.22)	-6.22(-9.10, -3.24)	0.00(0.00, 0.00)	26.89(20.99, 33.96)	-12.01(- 16.86, - 6.88)
Georgia	0.00(0.00, 0.00)	0.00(0.00, 0.00)	-1.29(-7.42, 5.24)	0.00(0.00, 0.00)	0.00(0.00, 0.00)	-20.38(- 42.50, 10.26)
India	628.85(285.59, 1749.55)	91.00(3.17, 329.10)	-5.61(-6.55, -4.66)	3711.2(2274.31, 6217.12)	298.19(112.14, 778.45)	-7.11(-8.04, -6.17)
Indonesia	152.81(10.87, 1027.76)	28.46(5.41, 115.30)	-4.81(-6.15, -3.45)	1483.2(606.44, 3172.18)	411.01(350.78, 480.45)	-4.93(-5.52, -4.34)

Iran	50.06(3.09, 356.16)	0.32(0.21, 0.45)	-16.31(-17.77, -14.82)	504.59(398.59, 617.75)	1.15(1.10, 1.17)	-26.38(-29.04, -23.61)
Iraq	4.73(3.84, 5.95)	0.00(0.00, 0.00)	-17.67(-24.68, -10.00)	100.73(72.84, 136.34)	0.00(0.00, 0.00)	-37.76(-25.94, -47.69)
Kyrgyzstan	0.00(0.00, 0.00)	0.00(0.00, 0.00)	-18.73(-29.47, -6.34)	0.00(0.00, 0.00)	0.00(0.00, 0.00)	-11.17(-36.96, 25.17)
Lao People's Democratic Republic	149.38(68.53, 333.98)	8.25(5.47, 11.91)	-8.14(-10.64, -5.58)	1922.05(1534.11, 2370.33)	118.76(99.23, 140.24)	-8.91(-10.71, -7.08)
Malaysia	37.04(17.10, 83.61)	0.05(0.03, 0.07)	-19.46(-21.51, -17.35)	689.65(628.63, 753.33)	0.00(0.00, 0.00)	-21.47(-23.75, -19.13)
Myanmar	551.87(232.42, 1410.03)	47.32(29.75, 108.55)	-3.63(-6.69, -0.46)	2287.79(1875.58, 2772.61)	356.12(300.79, 420.63)	-3.50(-5.53, -1.42)
Nepal	45.14(28.31, 66.79)	2.12(1.17, 3.69)	-11.14(-13.07, -9.17)	778.43(632.09, 952.82)	8.36(7.19, 9.65)	-15.21(-17.23, -13.15)
Oman	34.57(5.93, 143.67)	1.38(0.93, 1.99)	-12.38(-17.32, -7.14)	262.62(52.88, 886.68)	2.43(0.02, 18.12)	-17.87(-24.28, -10.92)
Pakistan	526.06(22.30, 3573.74)	239.57(21.51, 671.50)	-2.45(-3.71, -1.16)	4642.11(818.76, 14315.12)	1682.59(1333.63, 2061.65)	-2.64(-3.38, -1.89)
Philippines	61.81(4.85, 290.82)	1.36(0.12, 5.43)	-9.26(-10.37, -8.13)	429.82(349.69, 511.96)	12.23(10.30, 14.41)	-11.31(-12.22, -10.38)
Republic of Korea	4.63(3.71, 5.69)	0.10(0.08, 0.13)	-11.86(-14.41, -9.24)	3.75(1.55, 7.23)	0.76(0.19, 2.03)	-7.03(-9.32, -4.68)
Saudi Arabia	28.56(9.19, 57.02)	1.00(0.65, 1.41)	-12.46(-14.69, -10.16)	102.09(102.09, 102.09)	0.48(0.02, 2.25)	-21.66(-25.24, -17.91)
Sri Lanka	234.85(103.91, 549.05)	0.00(0.00, 0.00)	-32.82(-36.13, -29.35)	4875.94(4378.65, 5412.70)	0.00(0.00, 0.00)	-37.99(-42.68, -32.92)
Syrian Arab Republic	2.27(1.34, 3.74)	0.00(0.00, 0.00)	-16.14(-24.64, -6.67)	2.84(2.21, 3.65)	0.00(0.00, 0.00)	-40.02(-51.72, -25.47)
Tajikistan	300.29(34.60, 880.73)	0.00(0.00, 0.00)	-30.47(-36.60, -23.74)	757.22(81.11, 2882.08)	0.00(0.00, 0.00)	-29.36(-39.20, -17.94)
Thailand	155.38(70.98, 305.88)	0.56(0.13, 0.86)	-15.08(-16.78, -13.35)	1518.48(1357.07, 1704.08)	8.51(7.33, 9.83)	-14.07(-15.50, -12.62)
Timor-Leste	577.78(109.27, 1774.58)	0.00(0.00, 0.00)	-19.22(-26.71, -10.96)	8658.48(795.09, 38601.10)	0.00(0.00, 0.00)	-20.68(-27.66, -13.03)

Türkiye	4.14(3.37, 5.09)	0.00(0.00, 0.00)	-7.57(-9.79, -5.30)	19.68(12.45, 29.77)	0.00(0.00, 0.00)	-22.06(-28.50, -15.03)
Turkmenistan	0.00(0.00, 0.00)	0.00(0.00, 0.00)	-5.20(-5.97, -4.42)	0.00(0.00, 0.00)	0.00(0.00, 0.00)	-11.93(-26.27, 5.21)
Uzbekistan	4.33(3.50, 5.25)	0.00(0.00, 0.00)	-16.20(-23.42, -8.30)	0.07(0.03, 0.26)	0.00(0.00, 0.00)	3.16(-12.89, 22.16)
Viet Nam	71.42(12.26, 295.97)	0.27(0.18, 0.41)	-14.51(-16.67, -12.30)	232.24(74.44, 573.04)	1.25(1.03, 1.48)	-13.06(-14.87, -11.21)
Yemen	2826.06(852.94, 6689.82)	1641.84(318.61, 3882.07)	-1.98(-3.13, -0.82)	11242.20(3785.13, 25628.26)	6391.84(5059.80, 7910.35)	-2.73(-4.45, -0.98)
Global	965.67(502.56, 1898.07)	806.00(318.93, 1570.18)	-1.17(-1.57, -0.77)	3689.81(3133.05, 4440.58)	3485.27(2804.46, 4435.69)	-0.46(-0.65, -0.27)
Asia	280.02(103.98, 923.97)	62.70(7.60, 191.61)	-4.35(-5.05, -3.65)	1596.76(1127.96, 2405.77)	325.46(254.12, 477.93)	-4.85(-5.20, -4.50)
High SDI	0.83(0.47, 1.39)	0.05(0.03, 0.07)	-10.28(-11.35, -9.20)	1.94(1.82, 2.12)	0.06(0.01, 0.16)	-14.17(-15.94, -12.36)
High-middle SDI	6.25(1.02, 42.80)	0.74(0.14, 1.93)	-7.31(-9.05, -5.54)	56.87(19.76, 218.36)	6.41(2.50, 16.81)	-7.90(-9.95, -5.81)
Middle SDI	297.44(134.69, 690.53)	177.62(70.05, 334.71)	-2.15(-2.64, -1.66)	1209.18(978.96, 1583.60)	750.12(489.97, 1093.02)	-2.16(-2.58, -1.74)
Low-middle SDI	978.75(448.70, 2297.63)	630.94(249.10, 1273.67)	-1.90(-2.31, -1.48)	5337.13(4276.45, 6839.83)	3160.63(2438.56, 4089.91)	-2.11(-2.30, -1.91)
Low SDI	4811.47(2580.65, 8569.12)	2869.17(1107.42, 5683.87)	-2.17(-2.45, -1.89)	17529.12(14625.05, 20999.26)	11883.52(9755.64, 14691.05)	-1.43(-1.60, -1.27)

ASIR age-standardized incidence rate, *ASR* age-standardized rate, *DALY* disability-adjusted life year, *EAPC* estimated annual percentage change, *UI* uncertainty interval

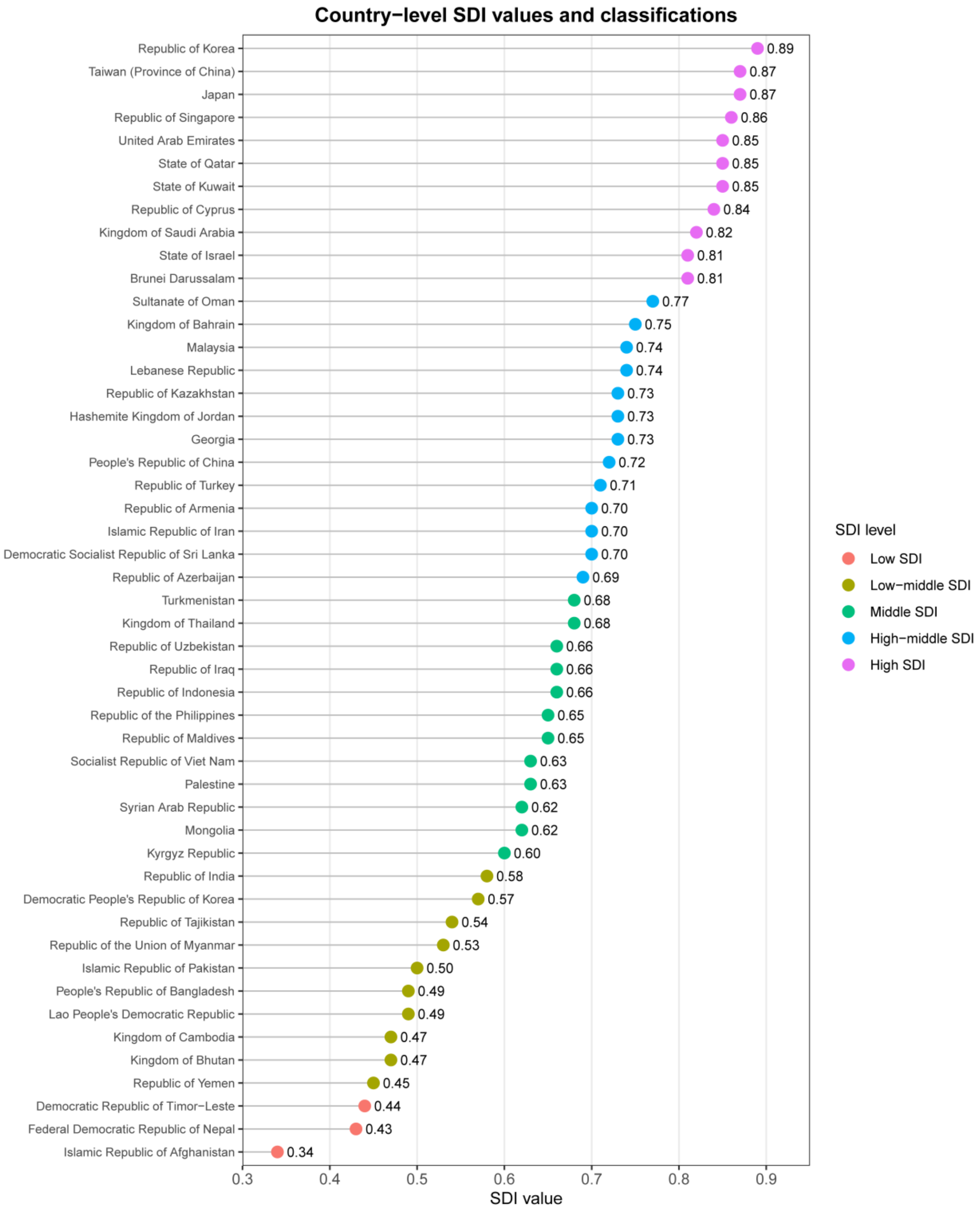

Figure S1. Country-level SDI values and classifications of the 49 Asian countries included in this study.

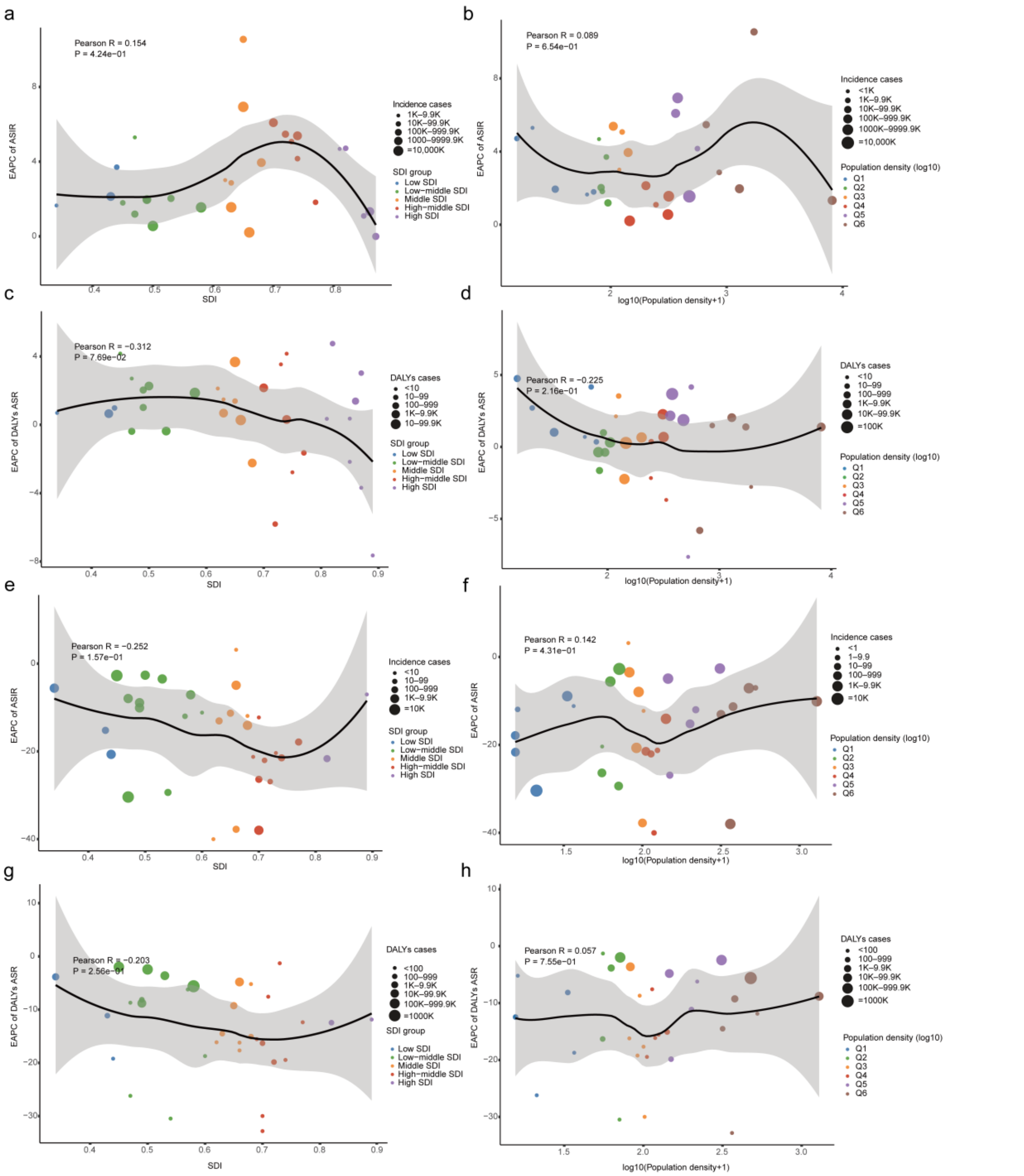

Figure S2. Correlation analyses between estimated annual percentage change (EAPC) and Socio-demographic Index (SDI) or population density.

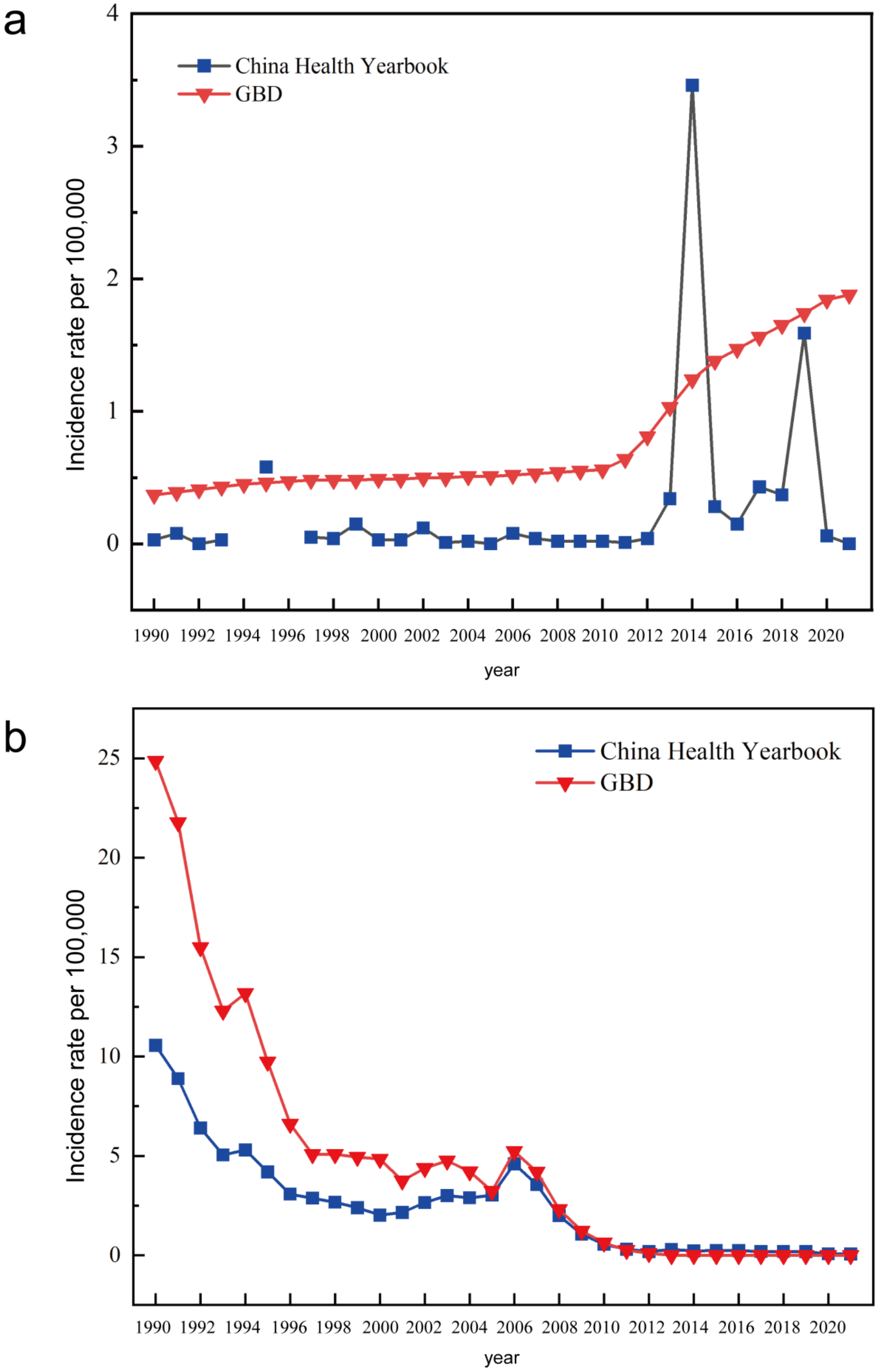

Figure S3. Comparison of incidence rate per 100,000 people in China Health Yearbook and GBD from 1990 to 2021.
